# A rare presentation of an elderly patient with acute lymphocytic leukemia and platelet count of zero associated with ST-elevation myocardial infarction, pulmonary thromboembolism in the setting of SARS-CoV 2: a case report

**DOI:** 10.1186/s43044-021-00162-9

**Published:** 2021-05-01

**Authors:** Arash Hashemi, Fady Gerges, Haseeb Raza Naqvi, Irina Kotlar, Sara Moscatelli, Ashkan Hashemi, Yasmin Rustamova, Abdallah Almaghraby

**Affiliations:** 1 Department of Cardiology, Erfan General Hospital, Tehran, Iran; 2 Department of Cardiovascular Science, Mediclinic Al Jowhara Hospital, P.O. box 84142 Al Ain, United Arab Emirates; 3 Department of Cardiac Electrophysiology, Mukhtar A. Sheikh Hospital, Multan, Pakistan; 4 Cardiology Department, University Clinic of Cardiology, Skopje, North Macedonia; 5 Pediatric Cardiology Services, Royal Brompton Hospital and Harefield NHS Foundation Trust, London, UK; 6 Department of Cardiology, Sina Hospital, Tehran University of Medical Sciences, Tehran, Iran; 7 Department of Cardiology, Azerbaijan Medical University, Baku, Azerbaijan; 8 Department of Cardiology, University of Alexandria, Alexandria, Egypt

## Abstract

**Background:**

Novel coronavirus disease 2019 (COVID-19) is known to lead not only to severe acute respiratory syndrome, but also can result in thromboembolic events in both the venous and the arterial circulation by inducing coagulation disorders. The potential causes of coagulopathy are inflammation, platelet activation, endothelial dysfunction, and stasis. The thrombotic events including pulmonary embolism, deep venous thrombosis as well as intracatheter thrombosis are more likely to develop in patients infected with severe form of SARS-CoV-2 who are admitted to ICU. Furthermore, these events contribute to multi-organ failure.

**Case presentation:**

Herein, we report a case of an immunocompromised COVID-19 elderly patient with acute lymphocytic leukemia who developed myocardial infarction with ST elevation in the setting of acute pulmonary thromboembolism in the presence of zero platelet count. Despite successful urgent coronary revascularization and platelet transfusion, the patient eventually died after failed resuscitation efforts.

**Conclusion:**

Patients with COVID-19 infection are at a greater risk of developing cardiovascular complications, but their appropriate management can decrease the risk of fatal events. Coronary thrombosis associated with pulmonary thromboembolism in the setting of thrombocytopenia is a rare and a complex to manage condition. Significance of single antiplatelet agent in STEMI with thrombocytopenia merits further studies. According to expert opinions and literature reviews, we must avoid dual antiplatelet therapy in these patients and keep platelet transfusion as a standard therapy to avoid drastic bleeding complications.

## Background

COVID-19 patients with associated venous thromboembolism can eventually develop STEMI requiring urgent revascularization. Despite this fact, the presentation of patients with STEMI during pandemic time was reduced, probably due to quarantine and social isolation. The most frequently reported abnormalities in coagulation profile in these patients are increased D-dimer, thrombocytopenia, and prolongation in thrombin time. Severe thrombocytopenia furthermore worsens the prognosis and suggests the presence of additional etiologies. Coexistence of all three patterns in a COVID-19 patient is a rare condition. Overlapping of clinical manifestations additionally complicates the initial diagnosis.

## Case presentation

An elderly 70-year-old female patient was hospitalized for receiving her regular chemotherapy dose and with no specific complaints.

She reports history of diabetes mellitus (DM), hypertension (HTN), and on chemotherapy treatment for acute lymphocytic leukemia (ALL) including vincristine and idarubicin.

During admission, regular PCR screening for SARS-CoV-2 came positive.

On day two of admission, patient developed dry cough and chest pain and acute dyspnea.

On physical examination, patient was afebrile, however, looked weak and fragile with dyspnea NYHA class III. She had mild petechiae and ecchymosis on lower limbs.

Blood pressure was 105/70 mm Hg, pulse was equal on both sides with heart rate of 90 bpm, respiratory rate 30/min, and oxygen saturation (SpO_2_) 92% at room air.

There were no cardiac murmurs on cardiac auscultation.

Chest examination revealed coarse crackles on both lungs.

**Fig. 1:**
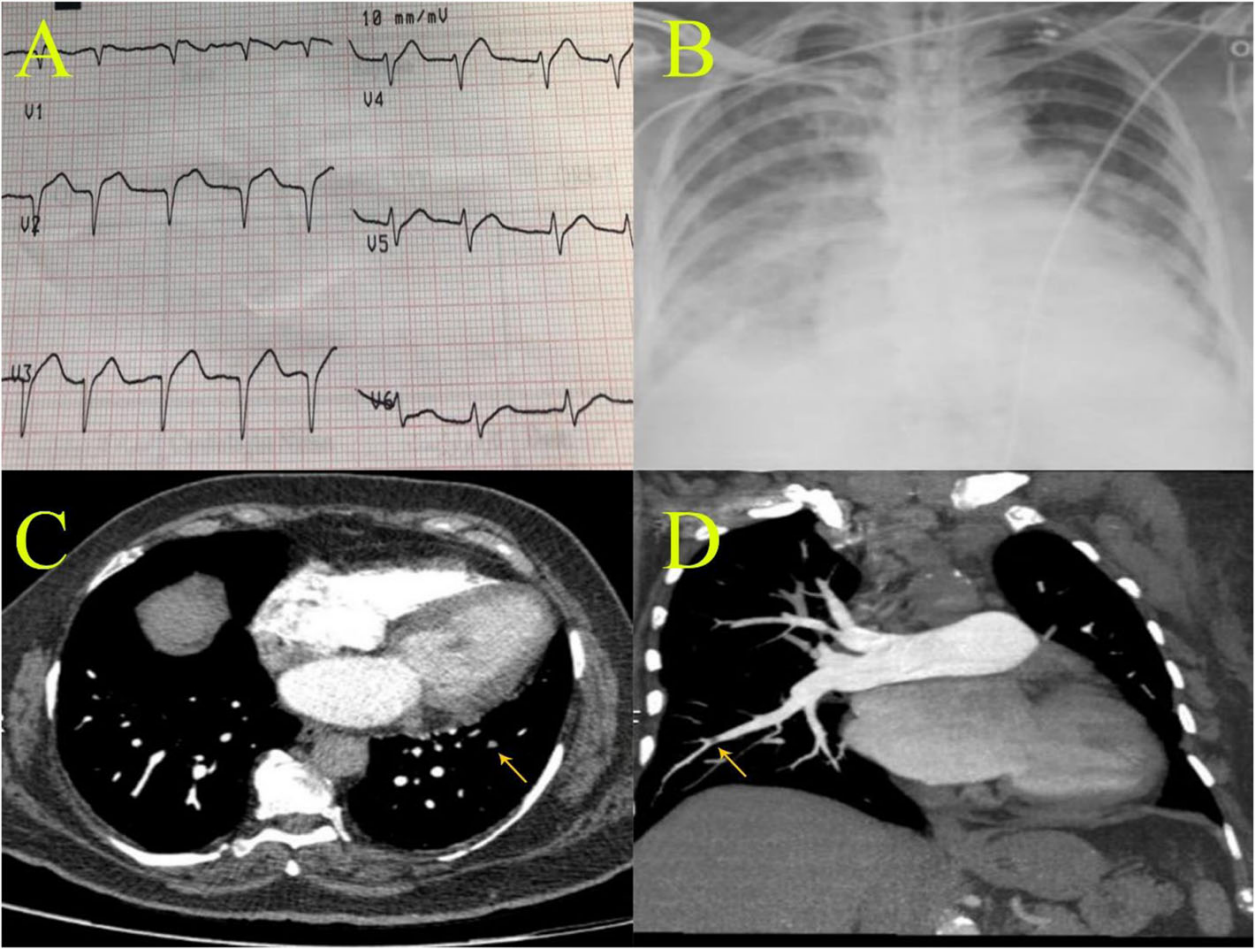
(Panel **a**) ECG showing atrial fibrillation rhythm and Q waves with residual ST elevation in the anterior chest leads, (panel **b**) chest X-ray showing increased cardiac shadow with bilateral lung infiltrations with classic ground glass opacities, (panels **c** and **d**) MSCT pulmonary angiography showing a picture of acute pulmonary thromboembolism (PTE) involving subsegmental branches of both lower lung lobes (yellow arrows)

Investigations including lab and imaging were as follows:

- Electrocardiogram (ECG) showed normal sinus rhythm with a rate of 95 bpm
- Computed tomographic pulmonary angiography (CTPA) showed picture of acute pulmonary thromboembolism (PTE) involving subsegmental branches of both lower lung lobes. CT depicted subpleural ground glass opacities as well on both lungs (Fig. 1).
- Fasting blood sugar, 189
- Serum creatinine, 0.95 mg/dL
- Creatine phosphokinase, 500 IU/L
- Creatine kinase myocardial band (CK-MB), 75 IU/l
- Serum potassium (K), 4.4 mEq/L
- Serum sodium (Na), 138 mEq/L
- Prothrombin time (PT), 17 s (normal 12–14 s)
- Partial thromboplastin time (PTT), 48 s (normal 25–35 s)
- International normalized ratio (INR), 1.5
- Hemoglobin, 8 g/dl
- WBC, 2.0 × 1000/Ul
- Hematocrit, 24 %
- Platelet count (PLT), 0 × 10^9^ per/L (recorded zero by lab)
- Lymphocytes, 61%
- COVID-19 IgM 0.10 (positive >1.1)
- COVID-19 IgG 3.45 (positive >1.1)
- D-dimer >10,000 μg/L
- Echocardiography (Echo): mild left ventricular (LV) dysfunction with LV ejection fraction (EF) 55%. Mildly enlarged right ventricle with normal pulmonary pressure. No resting wall motion abnormality.
- Pelvi-abdominal ultrasound abdomen: dilated inferior vena cava 24 mm with right kidney larger than normal with a size of 145 mm.
- Chest X-ray: bilateral lung infiltrations with classic ground glass opacities.

Cardiac and hematologic team together decided not to start anti-coagulation for her PTE because the patient’s platelet (PLT) count was 0 × 10^9^ per/L after repeating twice, supposedly due to chemotherapy-induced thrombocytopenia (CIT); however, disseminated intravascular coagulation (DIC) cannot be ruled out especially in presence of high D-dimer and prolonged PT and aPTT. In addition, patient was hemodynamically stable, and hence, we have not opted for anti-coagulation.

Patient started to be hypoxic with SpO_2_ 89%; however, blood pressure was maintained at normal values. Hypoxia improved on supplemental oxygen.

Urgent transfusion of random donor platelets and fresh frozen plasma was done.

On day three of admission, patient developed excruciating central chest pain with raised ST segment on ECG.

Via a trans-radial access through a 4-French size sheath, primary percutaneous coronary intervention (PCI) to left anterior descending artery (LAD) was performed.

Before PCI, elective endotracheal intubation was done to prevent nasogastric blood aspiration resulted from iatrogenic severe epistaxis induced by nasogastric tube insertion.

Our priority during PCI was to achieve successful revascularization through performing only plain old balloon angioplasty (POBA) without using a coronary stent to avoid starting dual antiplatelet therapy (DAPT) for the patient in view of her severe thrombocytopenia.

However, after multiple runs of thrombosuction to remove the heavy thrombus burden followed by POBA, we encountered abrupt reclosure of the LAD artery and no-reflow phenomenon with continuous reformation of coronary thrombi. Accordingly, we had to deploy the newest second-generation drug-eluting stent (DES) with optimal results and TIMI (thrombolysis in myocardial infarction) grade III flow with full resolution of chest pain.

Patient received total of 20 units of platelets, after which PLT count improved to 20 × 10^9^ per/L.

During PCI, patient developed rapid atrial fibrillation (AF) that responded successfully to 150 mg intravenous bolus dose of amiodarone with reversion to sinus rhythm.

INR was 1.5, and active clotting time (ACT) during PCI was 381 s without anti-coagulants which posed an extremely high risk of bleeding. Hence, no anti-coagulant was given during or after the PCI procedure.

Medications during admission were as follows:

- Remdesivir loading dose of 200 mg IV infused over 30–120 min on day 1, then day 2 and thereafter, 100 mg IV qDay for total of 7 days.
- Meropenem, insulin, ticagrelor, carvedilol, amiodarone, amlodipine, furosemide, N-acetyl cysteine, oral hypoglycemic agents, fentanyl drip, pantoprazole, fresh frozen plasma, random donor platelets transfusion.

During coronary care unit admission, we decided to start ticagrelor as a single anti-platelet agent post-PCI after 180 mg of a loading dose given before PCI.

LVEF improved to 35% compared to 20% before PCI.

After ensuring all weaning criteria are present, patient was extubated after 3 days and was more stable in terms of hemodynamics and oxygen saturation.

Moreover, LVEF further improved to 50% on screening Echo 3 days later. And PLT count increased to 25 × 10^9^ per/L.

However, on day six, the patient’s condition quickly deteriorated due to acute severe respiratory failure with hypoxia.

The patient was intubated and mechanically ventilated. However, she had cardiac arrest with pulseless electrical activity and then asystole. Cardiopulmonary resuscitation (CPR) could not revive the patient, and she died.

## Discussion

Cancer patients receiving chemotherapy have a seven-fold higher risk of venous thromboembolism compared with non-cancer patients; furthermore, the estimated rate of thrombocytopenia induced by chemotherapeutic agents can vary between 21 and 70% [1].

In the era of the COVID-19 pandemic, we as physicians are faced with common cardiovascular adverse events including acute myocardial infarction, venous thromboembolism, heart failure, myocarditis, and arrhythmias. Patients with cancer are particularly susceptible to the virus because of the immunosuppressive state, with greater risk from severe COVID-19 illness and death [2].

Furthermore, vascular and systemic inflammation induced by SARS-CoV-2 infection may cause endothelial dysfunction and prothrombotic and hypercoagulable state. Thrombocytopenia due to myelosuppressive chemotherapy needs to be taken into consideration prior to initiation of anticoagulation. By far, it is also known that the virus itself is associated with drop in PLT count [3]. Moreover, the risk of bleeding particularly when PLT count drops below 20×10^9^ outweighs the potential benefit of anticoagulant therapy. While large randomized trials for treatment of venous thromboembolism (VTE) and concomitant thrombocytopenia are lacking, expert opinion agrees on withholding anticoagulation and considering the insertion of vena cava filter when the level of PLT is critical [2].

On the other hand, it is quite rare to find a cancer patient presenting with acute coronary syndrome (ACS) and low platelet count. Patients with acute myocardial infarction and thrombocytopenia have a two-fold increase in in-hospital mortality and an increased risk of ischemic stroke, cardiogenic shock, and cardiac arrest [4].

Management of ACS in cancer patients with thrombocytopenia is a challenging domain. This challenge is even greater in this patient that has been diagnosed with COVID-19 complicated by acute pulmonary embolism. The use of antiplatelet drugs in patients with ACS and thrombocytopenia always raises a question. In such patients, aspirin administration may be used when platelet counts are >10,000/mL [5].

There is a clear medical need for strategies to lower the risk of bleeding in PCI patients, without losing ischemic protection. The results from the TWILIGHT sub-analyses offer important insights about ticagrelor as a monotherapy in these high-risk patients [6].

The standard dose of unfractionated heparin in patients undergoing percutaneous coronary intervention (PCI) in case of severe thrombocytopenia is 30–50 U/kg. Further dose adjustment can be done to keep activated clotting time (ACT) not less than 250 s [5]. The safety of antiplatelet therapy and PCI in patients who have ACS and thrombocytopenia is unknown because there are no randomized studies to suggest treatment approaches in such patients.

According to the guidelines provided by the American Society of Clinical Oncology, thrombocytopenic patients with the platelet count of 40,000 to 50,000/mL can undergo any invasive procedure, but in case of intraoperative or postoperative bleeding, platelets can be transfused accordingly [7]. Prophylactic platelet transfusion is not recommended in patients undergoing cardiac catheterization with thrombocytopenia, unless:

1. Platelet count <20,000/mL and one of the following: (a) high fever, (b) leukocytosis, (c) rapid fall in platelet count, (d) other coagulation abnormality.
2. Platelet count <20,000/mL in solid tumor patients receiving therapy for bladder, gynecologic, or colorectal tumors; melanoma; or necrotic tumors [8].

Regarding revascularization in patients presenting with acute myocardial infarction and thrombocytopenia, POBA should be reserved for patients with platelet count <30,000/ml. Balloon angioplasty might be the treatment of choice also because the duration of DAPT can be limited to 2 weeks. Bivalirudin and/or radial approach should be considered to minimize the risk of bleeding [5].

## Conclusions

The increased risk of thrombotic complications is well recognized in patients affected by the SARS-CoV-2 virus. Management of PTE and STEMI in COVID-19 patient with cancer and thrombocytopenia is extremely complex and challenging and is associated usually with a grave prognosis.

Patients who suffer from malignant diseases are more prone to develop severe form of infection and complications from SARS-CoV-2 virus. Treatment of venous and arterial thrombosis in cancer patients with thrombocytopenia poses a challenge.

Further studies are expected to answer the questions regarding the optimal anticoagulant and anti-platelet for these patients’ subsets.

## Data Availability

The data is available for sharing.

## Abbreviations

ACS: Acute coronary syndrome
ACT: Active clotting time
AF: Atrial fibrillation
ALL: Acute lymphocytic leukemia
CIT: Chemotherapy-induced thrombocytopenia
CK-MB: Creatine kinase myocardial band
CPR: Cardiopulmonary resuscitation
CTPA: Computed tomographic pulmonary angiography
DAPT: Dual antiplatelet therapy
DES: Drug-eluting stent
DIC: Disseminated intravascular coagulation
DM: Diabetes mellitus
ECG: Electrocardiogram
Echo: Echocardiography
EF: Ejection fraction
HTN: Hypertension
INR: International normalized ratio
K: Potassium
LAD: Left anterior descending artery
LV: Left ventricular
Na: Sodium
PCI: Percutaneous coronary intervention
PLT: Platelet count
POBA: Plain old balloon angioplasty
PT: Prothrombin time
PTE: Pulmonary thromboembolism
PTT: Partial thromboplastin time
SpO_2_: Oxygen saturation
TIMI: Thrombolysis in myocardial infarction
VTE: Venous thromboembolism
